# Involvement in family planning service utilization and associated factors among married men at Debre Tabor town, Northwest Ethiopia, 2017

**DOI:** 10.11604/pamj.2021.38.211.22470

**Published:** 2021-02-24

**Authors:** Tadesse Wuletaw Demissie, Enatinesh Mesfin Tegegne, Araya Mesfin Nigatu

**Affiliations:** 1 Department of Nursing, Debre Tabor Health Science College, Debre Tabor, Ethiopia,; 2 Department of Nursing, Debre Tabor University, Debre Tabor, Ethiopia,; 3 Department of Health Informatics, Institute of Public Health, University of Gondar, Gondar, Ethiopia

**Keywords:** Men involvement, family planning, Debre Tabor

## Abstract

**Introduction:**

men´s involvement in family planning (FP) can be either as a user of male contraceptive methods and/or support of the male partners. In some developing countries, levels of communication on this issue is low for decision-making. Unmet need for FP suggested that unwanted pregnancy and unsafe abortion are the main causes of maternal mortality in Ethiopia. Men’s involvement in family planning is very important to improve women´s health in particular and reproductive health in general.

**Methods:**

the community based cross-sectional study design was conducted to assess men´s involvement in family planning service and associated factors among married men at Debre Tabor town. A simple random sampling method was used to include 382 married males. Data were collected by face-to-face interview using a structured questionnaire. The data were entered into Epi Info 7 and were analyzed using SPSS version 21 statistical software package.

**Results:**

from three hundred and eighty-two participants, 373 participated yielding a 97.6% response rate. The age range of the participants’ was from 20 to 65 years, the mean age was 38.6 with the standard deviation of ±7.8. The majority of the participants were Orthodox followers 358 (96%). About 33.2% of them were greater than secondary education level. The magnitude of male involvement in family planning was 254 (68.1%), 370 (99.2%) of the participants had information on different family planning methods. Adjusting all other factors for the final model, educational status AOR = 2.39 [1.084, 5.260], source of information AOR [95%CI] = 1.88 [1.016, 3.485], men’s approval AOR [95%CI] = 0.07 [0.036, 0.134], ever used contraceptive AOR [95%CI] = 0.21 [0.064, 0.705] were found to be associated with men’s involvement.

**Conclusion:**

the level of male involvement was moderate, but their actual utilization is low.

## Introduction

Men´s involvement in reproductive health has two major sides, as men give sufficient support in needs, choices and rights to their partners in reproductive health and fertility control, on the other hand, men´s owns reproductive health issues related to knowledge, contraceptive use and safe sexual behavior [1]. Family planning has got acknowledgment in the 1990s by many women´s health programs and must be viewed in the broader context of reproductive health; the International Conference on Population and Development (ICPD) held in Cairo 1994 noted that special efforts ought to be made to emphasize men´s shared responsibility and promote their active involvement in responsible parenthood, sexual and reproductive behavior, including family planning [2]. Men´s participation in family planning includes, encourage and support their partners (wives) in contraception and encourage peers to use family planning and influence the policy environment to be more conducive for male-related programs [3]. Available studies showed that in many developing countries males often dominate in making important decisions in the family, which include those concerned with reproduction, family size, and contraceptive use [4].

Spousal communication on contraception and reproductive goals suggests that the couple has an egalitarian relationship. Studies have shown that couples who discuss the number of children they desire or the use of family planning are more likely to use a contraceptive and achieve their reproductive goals than those who do not have communication with each other [5]. Men have the final say in decision making about family size and the use of contraceptives [6]. A considerable discordance between spouses on questions of family planning and desired family size has also been identified; in some developing countries, levels of communication on these topics are low [7]. African men are not only head of the household but also are overall responsible for families; men have more influence on reproductive decision since they typically control the family asset [8]. In Ethiopia, FP was initiated four decades ago; however, even after such a long period, the service has been amongst the lowest in Africa with 42% contraceptive prevalence rate (CPR) and 18.8% unmet need for FP with current time [9]. Several factors are incriminated for the low coverage of FP services including the desire to have more children, lack of knowledge about contraceptive use and where to find contraceptives, health concerns, religious prohibition, husband opposition and low involvement of males [10]. Men involvement in family planning could improve reproductive health and gender issues [11], but males involve as simply partners. Even though there are country and regional variation in family planning users and methods, male´s cooperation has been remained steady over the past few decades [12]. Therefore, this study will show the level and role of male involvement in family planning; furthermore, describe factors associated with male involvement in family planning practice.

## Methods

The community-based cross-sectional study design was employed to assess men´s involvement in family planning service utilization and associated factors among married men.

**Settings**: the study was conducted at Debre Tabor town. Debre Tabor town administration is one of the 13 districts and five town administrations found in South Gondar administrative zone, Ethiopia. It is located 105km away from Bahir Dar and 666 km away from Addis Ababa. Debre Tabor town is additionally divided into 4 kebeles (the smallest administrative boundary of the Town). The town has a total population of 83,082 of whom 39,781 are males and 43,301 are females (BOFED 2015). The total number of households in the town is 13200 and there is 1 governmental Hospital, 3 governmental health centers, 3 private clinics, and 2 private pharmacies. The study was conducted from April to June 30/2017 in Debre Tabor town administration of South Gondar, Ethiopia.

**Study population and sampling**: all married men who are living at Debre Tabor town administration for the last six months. The sample size was calculated using a single population proportion formula. An estimated 65.5% proportion of male involvement [13] with a 95% confidence level, 5% marginal error and considering 10% none response rate were considered; then the final sample was 382. Debre Tabor town has four kebeles. Currently, there are about 13200 households in the town with 4624 married males. A simple random sampling (lottery method) technique was undertaken to select the households. The four kebeles were included in the study. Participants were allocated proportionally to get 382 married males from 4624 married males in the four kebeles. Married males were selected systematically every 12 households in each kebele.

### Operational definition

**Current use of contraceptive**: those respondents who are using the contraceptive method or whose partners are using the contraceptive method during the data collection period.

**Men involvement**: spousal communication, approval, support of the husband and both partners usage of contraceptive and score above the mean.

**Married man**: a man who lived together with a woman with a legal relationship for greater than 6 months.

**Good men´s attitude towards family planning**: respondents who answered agree and above on the attitude questions.

**Data collection procedures and instruments**: data were collected using a structured questionnaire with face-to-face interview technique.

**Data management and analysis**: the data were entered using Epi Info version 7; SPSS version 21 was being used for data cleaning and analysis.

**Ethical approval and consent to participants**: ethical clearance was obtained from Debre Tabor University Ethical Review Committee, permissions were being taken from the South Gondar Zonal Health Department. The necessary explanation about the purpose of the study and its procedure was given to the participants and then, informed verbal consent was obtained from the respondents. The study participants had got information about that, they have the full right not to participate in the study if they are not willing. Anonymity was explained clearly for the participant to ensure confidentiality.

## Results

**Socio-demographic characteristics**: from 382 participants invited to participants, 373 participants were participated in the study yielding 97.6%. The age of the participants ranged from 20-65 years, the mean age was 38.6 with the standard deviation of 7.8. The majority of the participants were Orthodox, 359 (96.2%) and 11 (2.2%) were Muslims. Concerning educational status most participants 124 (33.2%) were greater than secondary education, 11 (2.9%) of the participants were unable to read and write and with regard to occupation 163 (43.7%) were governmental employees, 22 (5.9%) of them were unemployed and farmers took the least rank 9 (2.4%). The highest frequency of age at marriage is 30 years 130 (34.9%) and most of the respondents 122 (32.7%) have 3-4 children and the least 41 (11%) of them have more than five children (Table 1). Married men had information on family planning methods by different media for them and their spouses; 99.2% of the participants had information on different family planning methods; Parents took the first rank to be a source of information and television accounts the least (Table 2).

**Table 1:** frequency distribution of socio demographic characteristics among married men at Debre Tabor town, Northwest Ethiopia, 2017

Variable	Frequency (n=373)	Prevalence (%)
**Age of respondents**		
20-24	4	1.1
25-29	32	8.6
30≥	337	90.3
Ethnicity		
Amhara	373	100
**Religion**		
Orthodox	359	96.2
Muslim	11	2.9
Adventist	3	0.8
**Educational status**		
Unable to read and write	11	2.9
Able to read and write	30	8
Primary school	117	31.4
Secondary school	91	24.4
Higher education	124	33.2
**Occupation**		
Farmer	9	2.4
Governmental employee	163	43.7
Merchant	132	35.4
Unemployed	22	5.9
Others	47	12.6
**Age at first marriage**		
15-19	24	6.4
20-24	100	26.8
25-29	119	31.9
>30	130	34.9
**Number of children**		
None	97	26
1-2 children	113	30.3
3-4 children	122	32.7
Greater than 5 children	41	11

* **Others**: students, daily laborers

**Table 2:** frequency distribution of sources of information about family planning methods among married men at Debre Tabor town, Northwest Ethiopia, 2017

Variable	Frequency (n=373)	Prevalence (%)
Radio	169	45.3
Television	55	14.7
Health workers	127	34
Poster	335	89.8
Newspaper	336	90.1
Parents	369	98.9

**Men´s attitude and/or support towards contraceptives**: among the study participants, 358 (96%) of them had a good attitude or positive support on contraceptive utilization for them and their partners.

**Figure 1:**
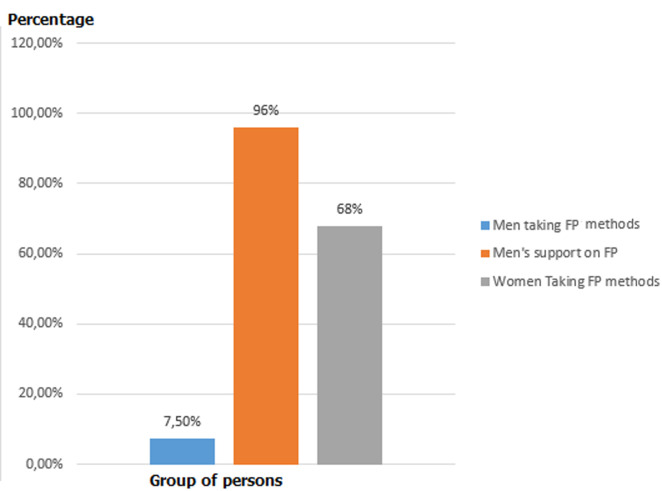
men’s support and partner’s actual taking of family planning (F/P) methods

**Men´s involvement in family planning**: men´s involvement on family planning was 254 (68.1%) 95%CI= [63.4%, 73.2%]. Most of the men had awareness on FP, support their spouse, 93.8% of them discuss about FP and 71.8% approve it, but only 28 (7.5%) of the men had used family planning; particularly 16 (4.3%) had used condom which is lower than the national prevalence in 2016 EDHS (11%) and 12 (3.2%) of them used abstinence and none of them used male sterilization. Sixty-eight percent of female partner took contraceptives which is higher than the national CPR (36% in 2016 EDHS); some of the reasons mentioned not taking contraceptives were: educational status of married men, source of information, knowing male type of contraceptive methods and previous experience of family planning methods (Figure 1). Even though married men had information, awareness, and support on family planning for them and their spouses, about 254 (68.1%) of men were involved in family planning.

**Factors associated with men´s involvement in family planning**: bivariate analysis was used to explore the association between men´s involvement in family planning with each of the determinant factors. Educational status of married men, sources of information about FP, know male type of contraceptive method, ever informed their partner to use FP, had desire to know more about FP methods, men´s approval for family planning method, men´s ever usage of FP method and previous usage of FP methods by female partner were met the minimum criteria (P<0.2) for further multivariate logistic analysis. Factors that had bivariate associations at p-value < 0.2 were then entered into multivariate logistic regression analysis. The variables associated by multivariate logistics analysis (p ≤ 0.05) with men´s involvement in family planning were: educational status of married men, sources of information´ about FP, married men ever informed their partner to use FP, had desire to know more about FP methods, men´s approved for family planning method, ever used of FP method by male partner and previously usage of FP methods by female partner (Table 3).

**Table 3:** bivariate and multivariate analysis for factors associated with men involvement in family planning methods among married men at Debre Tabor town, Northwest Ethiopia, 2017

Variable	Men involvement				Crude OR (95% CI)	Adjusted OR (95%CI)
			Not involved	Involved		
**Educational status**	Unable to read and write		5	15	0.33 [0.097, 1.151]	8.55 [2.118, 34.531]**
	Able to read and write		15	15	0.64 [0.216, 1.151]	2.57 [0.864, 7.639]
	Primary education		37	71	0.99 [0.322,3.012]	1.92 [0.908, 4.050]
	Secondary school		23	68	0.73 [0.247, 2.141]	2.39 [1.084, 5.260]*
	College and above		39	85	1	1
**Sources of information**	Radio	No	47	157	2.48 [1.587, 3.874]**	1.88 [1.016, 3.485]*
		Yes	72	97	**1**	1
	Health	No	89	157	0.55 [0.33, 0.886]*	0.80 [0.404, 1.571
	worker	Yes	30	97	**1**	1
	Poster	No	6	32	2.72 [1.103, 6.683]*	2.70 [0.856, 8. 487]
		Yes	113	222	1	1
**Know male contraceptive methods**	No		9	10	050 [0.198, 1267]	5.38 [0.117, 1.220]
	Yes		110	244	1	1
**Ever informed their partner to use FP**	No		36	44	0.48 [0.291, 0.803]**	2.34 [0.234, 23.360]
	Yes		83	210	**1**	1
					
**Desire to know more about FP**	No		37	47	0.48 [0.288, 0.790]**	0.23 [0.23, 2.250]
	Yes		82	209	1	1
**Approve for family planning method**	No		73	32	0.09 [0.054, 0.153]**	0.07 [0.036, 0.134]**
	Yes		46	222	1	1
**A man ever used family planning method**	No		115	213	0.81 [0.063,0.517]**	0.21 [0.064, 0.705]**
	Yes		4	41	1	1
**Female partner used FP method previously**	No		53	183	0.34 [2.118, 5.263]**	3.20 [1.752, 5.834]**
	Yes		66	69	**1**	**1**

NB:**p<=0.01, *<0.05 **FP**: family planning

## Discussion

The community-based cross-sectional study was conducted to assess the magnitude of married men´s involvement in family planning and associated factors at Debre Tabor town. In this study, the magnitude of married men´s involvement in family planning was 68.1% which is nearly in line with the study done in Bangladesh (63.2) [14] and Vietnam (63.7%) [15]. The current study was higher than the study done in West Pokot, Kenya (52%) [16], Turkey (30%) [17], only 4.8% were involved in Nigeria [18], in west Shewa zone, Ethiopia (36%) [19] and Afar, Ethiopia [20]. This could be due to governmental concern, economical factor, individual motivation and discussion and approval of the service by male partners. The result of the study is lower than the study conducted in rural Vietnam (74.4%) [21]. This could be due to less shared responsibility, educational status and poor attitude towards male participation in family planning service.

Participants who had used family planning were only 7.5% which is lower than the study conducted in Debre Markos town, Ethiopia (8.4%) in Eastern Tigray Ethiopia (15%)of the participants were using family planning methods directly [22,23], and in South-Eastern Turkey (39.6%) [24], but higher than the study conducted in Southern Ethiopia (5%) [13], in West Pokot Kenya (6%) [16]. This is due to awareness on family planning and accesses to family planning methods, economical, biological and socio-cultural impacts of men; furthermore, governmental and couple concerns on family planning methods. Based on access to information on family planning, about 99.2% married males had got information on family planning methods through different media which is consistent with the study done in Debre Markos, Ethiopia (99.2%) [22] but inconsistent with the study done in Malegedo Town, Oromia, Ethiopia (89%) [19]. This could be due to an access to mass media. This study result is higher than the study conducted in India (98% [25], in Southern Turkish 43.2% [24], in Ghana (89%) [26], in North Shewa, Ethiopia (93.9%) [27] in Woliyta Sodo Southern Ethiopia (96%) [13]. In Tigray Northern Ethiopia, 62.9% of the participants were informed about FP [23,26]. More involvement of health care providers to motivate, governmental concerns and media motivation to create awareness; furthermore, individual motivation could be mentioned.

### Factors associated with men´s involvement in family planning

According to this study, the multivariate analysis of logistic regression for men´s involvement in family planning pointed out that; educational status of married men, sources of information about FP, men ever informed their partner to use FP, had desire to know more about FP methods, men´s approval for family planning method, men´s ever usage of FP method and female partner used FP methods previously were found to be significantly associated with men involvement on family planning. The result pointed out that educational status of male partner was found to be one factor for men´s involvement in family planning; those respondents who were unable to read and write AOR [95%CI] = 8.55 [2.118, 34.531] and those in secondary education level AOR [95%CI] = 2.39 [1.084, 5.260] were 8.6 and 2.3 times more likely to be involved in family planning service respectively as compared to those in higher education level. This result is consistent with the study done in Eastern Tigray, Ethiopia [23], in Kenya [16] and Turkey [24]. This is because individual concern could not be affected by education, moreover, this could be the low economic status and motivation of the individuals. Source of information is also one significant factor. Radio as a source of information AOR [95%CI] = 1.88 [1.016, 3.485] was 1.8 times more likely affected men to be involved in family planning than those did not have information on the radio. This result is consistent with the study done in Malegedo Town, Oromia, Ethiopia [19].

In this study, men´s approval for family planning was strongly associated with men´s involvement in family planning. Those men approve family planning for their partner AOR [95%CI] = 0.07 [0.036, 0.134] were by 93% less likely to involve in family planning than those who did not approve family planning. This result is inconsistent with the study conducted in Bench Maji Zone, Ethiopia and Debre Markos, Ethiopia [28,29]. This may be due to the accessibility of information and shared responsibility; that only female partners were taking responsibility for family planning. The “ever used” family planning method was found to be a significant factor. Those male partners who used family planning AOR [95%CI] = 0.21 [0.064, 0.705] were by 79% less likely to be involved in family planning than those who did not use family planning. This could be due to the need for more children, less concern for discussion and disapproval of partner contraceptive usage. Previously usage of FP method was strongly associated with men´s involvement in family planning. Female partner used FP method previously AOR [95%CI] = 3.20 [1.752, 5.834] were 3.3 times more likely to be involved in family planning than those did not use FP methods previously. This may be the experience sharing for male partners and shared responsibility. Because family planning could be affected by many factors such as gender, biological factors, socio cultural aspects of both the community and the partner, policy of the country, decision making of the partners and communication, the study could point out the possible barriers and solutions to have consent on family planning or a partner by involving male partners.

## Conclusion

Governmental and non-governmental organizations, service providers, policymakers and relevant stakeholders should ensure availability, accessibility and sustainable advocacy for family planning services. The family planning programs should incorporate males in the uptake of family planning services. This could strengthen the service utilization and gender equality.

### What is known about this topic

- Male involvement is assumed to be the one factor for family planning;
- Male involvement in family planning is low due to socio-cultural, biological and economic reasons;
- Most female partners are using family planning methods without the male partner’s discussion and communication; this could affect the service utilization.

### What this study adds

- This study will add a body of knowledge on male involvement on family planning;
- This will help partner discussion and communication on family planning service;
- This study will be helpful for policy makers, program designers as well as for service utilizers.
